# Public Concern about the Sale of High-Caffeine Drinks to Children 12 Years or Younger: An Australian Regulatory Perspective

**DOI:** 10.1155/2015/707149

**Published:** 2015-10-04

**Authors:** Christina Mary Pollard, Catrina Lisa McStay, Xingqiong Meng

**Affiliations:** ^1^School of Public Health, Curtin University, Kent Street, GPO Box U1987, Perth, WA 6845, Australia; ^2^Department of Health in Western Australia, 189 Royal Street, East Perth, WA 6004, Western Australia, Australia; ^3^Flinders Centre for Innovation in Cancer, School of Medicine, Flinders University, Adelaide, SA, Australia

## Abstract

*Background. *Dietary exposure to high caffeine is a health risk for children. Governments are considering measures to restrict the sale of formulated caffeinated beverages (FCB) to children. *Objectives.* To investigate community concern about sales of high-caffeine drinks to children among Western Australian adults and describe Australian and New Zealand regulatory processes regarding FCB. *Methods.* Data from the 2009 and 2012 Department of Health's Nutrition Monitoring Survey Series of 2,832 Western Australians aged 18–64 years was pooled with descriptive and ordinal logistic regression analysis performed. Current regulatory processes for FCB are reported. *Results.* Most (85%) participants were concerned about the sale of high-caffeine drinks to children; 77.4% were very concerned in 2012 compared to 66.5% in 2009, *p* < .008. Females and those living with children had higher concern (odds ratio (OR) 2.11; 95% confidence interval (CI) 1.44–3.10; OR 2.16; 95% CI 1.51–3.09, resp., *p* < .001). Concern increased with each year of age (OR 1.04; 95% CI 1.02, 1.05, *p* < .001). *Conclusions.* Community concern regarding sale of high-caffeine energy drinks to children is high and increasing. Being female and living with children were associated with greater concern. These findings support the Australian and New Zealand regulatory controls of FCB, including labelling, promotion, and advertising to children.

## 1. Introduction

The growing concern regarding the consumption of caffeinated beverages such as energy drinks by children and adolescents has led to considerations of how to control or limit their intake by regulators and public health professionals [1–4]. Currently, there is no established safe level of consumption of caffeine for children and adolescents [5–7]. The National Health and Medical Research Council's Australian Dietary Guidelines, 2013, specify that high-caffeine beverages such as energy drinks are not suitable for children [8]. The American Academy of Paediatrics states “that caffeine and other stimulant substances contained in energy drinks have no place in the diet of children and adolescents” [9].

Reports of the adverse health effects of consuming an excess of energy drinks are increasing [10–13]. High acute consumption of caffeine increases the risk of toxic effects, particularly in children and adolescents who have not developed tolerance to caffeine [13, 14]. Clinical manifestations of caffeine toxicity include serious adverse cardiovascular effects, seizure, and deaths [14, 15]. Other effects include sleep disturbance, increased anxiety, nausea, palpitations, and headaches [6, 15, 16]. Childhood consumption may lead to habitual intakes in adult life [6]. Concomitant consumption of energy drinks and alcohol by youth has been identified as a public health issue of concern for health promoters, policy makers, and regulators [1, 12–14, 17, 18]. An additional concern is that many caffeinated products are essentially a soft drink, high in added sugar, nutrient poor, and energy dense, promoted for sale based on their stimulatory effects [19]. The displacement of nutrients from the diets of energy drink consumers and excess energy intake from discretionary foods contribute to obesity and related chronic disease, including cardiometabolic disease [8, 19].

Health promotion uses a combination of strategies to improve health of the population including environmental changes through food regulation [20]. The Australian Beverage Council asserts that energy drinks in Australia are stringently regulated [21]. Energy drinks are classified and regulated as a food under Standard 2.6.4-Formulated Caffeinated Beverages (FCB) of the Australia New Zealand Food Standards Code (the Code). This standard specifies that energy drinks contain between 145 mg/L and 320 mg/L of caffeine; comply to labelling provisions disclosing nutrient composition including caffeine content (per serving and per 100 mL) along with daily usage; and display warnings that product is not suitable for children, pregnant, or lactating women [22]. The United States (US) and Europe do not have an upper limit for caffeinated beverages [2]. The industry claims that the products are not marketed or promoted to children; however, half of sales are through the supermarket where there is no restriction on who purchases them [21].

Standard 2.6.4 of the Code was developed in 2001, amidst community concern regarding the availability of these caffeinated beverages to children [2]. Energy drinks, termed as FCB, are defined as “a non-alcoholic water-based flavoured beverage which contains caffeine and may contain carbohydrates, amino acids, vitamins and other substances, including other foods, for the purpose of enhancing mental performance” [22]. Although the concentration of caffeine in FCB is specified, the volume of a retail unit and therefore the amount of caffeine consumed per retail unit are not regulated. In addition to Standard 2.6.4, other standards regulate caffeine in food (Table 1).

Community opinion can inform regulatory process with consultation with key stakeholders, including the community, identified as “an instrumental component of the joint food regulation system, and is fundamental to the development of good food regulation policy” [23]. Assessing community attitudes and perceptions toward regulatory issues helps inform decision making.

This paper explores the attitudes of adults living in Western Australia to the sale of caffeinated beverages to children aged 12 years or younger. The aim was to measure the current level of community concern; assess changes between 2009 and 2012; and explore factors associated with attitudes including demographics and behaviour. This paper also describes the Australian and NZ regulatory decision making process and outcomes regarding FCB and other foods containing caffeine.

## 2. Methods

### 2.1. Surveys

Data from the Department of Health in Western Australia's (WA) statewide 2009 and 2012 Nutrition Monitor Survey Series (NMSS) were pooled for this analysis. Computer assisted telephone interviews of WA adults aged 18 to 64 years were conducted in July to August 2009 and 2012. The samples were randomly drawn from the Electronic White Pages for WA and stratified according to area of residence. All sample households with an address were sent a primary approach letter explaining the purpose of the survey, how the sample was selected, who would be asked to do the survey, and about how long it would take. Every household in the initial sample was called and asked if someone aged 18–64 years was resident and, if so, which one had the most recent birthday. No substitutes were accepted. The response rate (completed/contacted) was 81.6% and 82.4% for 2009 and 2012, respectively. Surveys were granted approval from the WA Department of Human Research Ethics Committee.

### 2.2. Measures

Participants were asked to rate how concerned they were about the sale of high-caffeine drinks to children 12 years or younger using a five-point Likert scale from one “not very concerned”; “somewhat concerned”; “neither unconcerned nor concerned”; and “quite concerned” to five points “very concerned.”

Their attitudes towards healthy eating were gauged by asking about the attention they paid to the health aspects of the food they eat, with the options of “pay a lot of attention,” “take a bit of notice,” or “don't really think about it.”

Demographic data collected were age, gender, education level, household income, employment status, country of birth, residential area, and living arrangement. Participants' self-reported height and weight were used to derive their body mass index.

### 2.3. Statistical Analysis

Data were pooled and weighted to account for sample design and post adjusted for age, sex, and geographic area of 2011 Estimated Resident Population of WA as it was the most recent census year. Descriptive statistics were used to report the prevalence of participants' concern about sales of high-caffeine drink to children 12 years or younger. Ordinal logistic regression on participants' attitude toward sales of high-caffeine drink was performed. The direction of the rating in the regression went from one “not very concerned” to five “very concerned.” A full model includes the following variables: survey year, demographics (gender, age group, education level, income, employment status, whether living with children, country of birth, and residential area), body mass index, and attention they paid to the health aspect of the food they eat. *p* values were derived from a survey design-based Pearson chi square test. Only variables with *p* value <.05 were retained in the final model and reported, with the exception of survey year which was retained in the model regardless of its significance. Survey module of Stata software version 12.0 (StataCorp LP, College Station, TX) was used for all analyses.

## 3. Results

A total of 2832 adults participated in the 2009 and 2012 surveys, and the sample details are shown in Table 2.

Table 3 shows that overall 85% of participants were “quite” or “very” concerned about the sales of high-caffeine drinks to children. Significantly more participants were “very concerned” in 2012 (77%) than in 2009 (67%), *p* < .008  (Table 3).

Regression analysis revealed female participants (OR 2.11) and those who live with children (OR 2.16) were twice as likely to be more concerned than their counterparts (Table 4, all *p* values <.05). Participants' concern also increased with age; for each incremental yearly increase in age participants were more likely to rate a high level of concern (OR 1.04). Participants residing in remote areas were significantly less likely to be very concerned than those living in other areas. There was no significant difference between participants' concern level across survey years when the other variables (body mass index and attention paid to the health aspect of food eaten) were included in the model.

## 4. Discussion

Community concern regarding the sale of high-caffeine drinks to children 12 years or younger remains high and increased between 2009 and 2012. As would be expected, females and participants living with children showed a higher level of concern. People in the community who are responsible for caring for children may be more aware of the availability of high-caffeine energy drinks to children, influences on children's dietary choices, and adverse impacts of energy drink ingredients.

Our findings show general population concern about sales of high-caffeine drinks to children, broader than US research which found that consumers of high-caffeinated beverages were concerned about product safety. A market research company assessed attitudes of consumers and nonconsumers of energy drinks in the US finding that 59% of those consuming energy drinks were concerned about possible adverse health effects [26]. Thirty-nine percent of people surveyed had reduced their intake due to perceived adverse health effects, and the majority supported the inclusion of maximum daily intake levels of caffeine information on the label (79% female; 71% male).

Our findings of high community concern are important as there has been a rapid growth in the number and types of caffeinated beverage products on the market. Between 2001 and 2010, energy drink sales in Australasia have quadrupled from 34.5 to 155.6 million litres and are predicted to reach 220 million litres by 2018 [2, 27]. The market leader, Frucor Beverages brand V, owned by Suntory Holdings, accounts for more than a third of sales volumes in Australasia. Red Bull (owned by Red Bull Australia Pty Ltd.) and Mother (owned by Coca-Cola Amatil) are also brand leaders, with Mother and V Australasian market specific brands [27]. The marketing of energy drinks is designed to appeal to young people, for example, edgy campaigns incorporating extreme sports [2, 28] such as the Red Bull Stratos promotion of Felix Baumgartner 9.09-minute fall from the stratosphere back to earth [29].

Regulatory organisations, researchers, and health promoters across the world are investigating the risks of energy drink consumption in vulnerable populations and there is national and international discourse on the risk of caffeine of consumption by vulnerable groups amidst increasing sales in the energy drink market. These risk assessments on energy drinks may help inform regulatory decision making in Australia. In 2010, Health Canada created the expert panel on caffeinated energy drinks to review the safety of caffeinated energy drinks in the food supply which concluded that although the risk of adverse health effects following the consumption of energy drinks in the Canadian context was low, serious adverse event signals had occurred [30]. The EFSA safety assessment of caffeine concluded that there was a lack of evidence on which to set a safe level of caffeine consumption for either children or adolescents [31]. The French Agency for Food, Environmental and Occupational Health & Safety (ANSES) concluded that a causal relationship between energy drink intake and adverse symptoms was assessed likely or very likely in 25 out of 212 analysed cases, reported to the agency since 2008 [10]. ANSES recommended that “at risk” individuals including children and adolescents should avoid consuming energy drinks and that energy drinks should not be consumed with alcohol or during exercise [10].

Globally, the consumption of caffeinated beverages amongst children and younger adolescents appears to be increasing [10, 17, 32]; however, there is a lack of current Australian and NZ dietary consumption trend data. The current risk assessment of caffeinated beverages in children used NZ intake data that is over 10 years old to guide policy options deliberations [2]. The estimated caffeine consumption derived from the 2002 NZ Children's National Nutrition Survey with the addition of one retail unit of an energy drink estimated that 70% of children and 40% of teenagers would exceed an adverse effect level of 3 mg/kg bw/day [7]. The 2012 Australian Health Survey estimated that the usual daily caffeine consumption in children 9–13 years was 23 mg per day for boys and 18 mg/day for girls; and the intake at the 95th percentile was 81 mg per day and 63 mg per day, respectively [33].

There is no established dietary reference standard, such as an acceptable daily intake (ADI) level, for caffeine intake in children or adolescents; however, ≤2.5 mg/kg body weight/day level has been used in risk assessments [6, 7, 10, 34]. In 2000, in a review of safety of dietary caffeine consumption including toxicological/pharmacological effects, addictive effects, or other hazards at low doses, the authors concluded that the “no effect” dose response, along with the threshold dose for behavioural effects in children, has yet to be established [6]. A recent systematic review of the health effects of energy drinks concluded that a precautionary approach was warranted until sufficient scientific evidence is available to establish safe levels of dietary consumption [5].

Consumption needs to be explored beyond mean intakes as there is evidence of high chronic and high acute caffeinated beverage consumption among children and adolescents. A survey across 16 European Union countries found that 18% of children and 68% of adolescents consumed at least one energy drink in the past year (average exposures were 1.01 mg/kg bw/day and 0.38 mg/kg bw/day, resp.) and energy drinks contributed on average 43% and 13% to total caffeine intakes [35]. However, this does not identify high or chronic consumption patterns. The proportion of children and adolescents assessed as “high chronic consumers” (minimum intake of 4-5 energy drinks per week) was 16% (average intake 0.95 L/week) and 12% (average intake 7 L/month), respectively. Of note, 12% of adolescents were also assessed as “high acute consumers” (energy drink intake ≥1.065 L/session).

In Australia the number of calls to the New South Wales Poison Centre in Australia regarding toxic effects experienced following energy drink exposure increased from 12 in 2004 to 65 in 2010, highlighting this issue of high acute consumption, with adolescents identified as being particularly at risk [12]. This led to the recommendation that energy drinks include the national poison hotline number on the energy drink label.

*The Australia and New Zealand Food Regulatory System and Response*. In Australia and New Zealand (NZ), the system of food policy and laws is founded by two key agreements. Firstly, the Food Regulation Agreement (Australia) between the Commonwealth and the States and Territories brings into operation a consistent and cooperative approach to food regulation across Australia. This agreement established several policy bodies: the Australia and New Zealand Food Regulation Ministerial Council (Ministerial Council), which became the Legislative and Governance Forum on Food Regulation (the Forum) in February 2011, along with the Food Regulation Standing Committee (FRSC). Secondly, the Joint Food Standards Treaty between Australia and NZ principally facilitates trade between these nations. These agreements are supported by the Food Standards Australia New Zealand Act 1991 (FSANZ Act), which established FSANZ as the statutory organisation charged with developing and administering the Code. The Food Regulatory System (FRS) separates policy, standards development, and implementation and enforcement decision making processes [23].

Provision of safe food for all Australians and New Zealanders, especially for vulnerable subgroups in the population, is a key tenet of the FRS. Under the FSANZ Act, the objectives (in descending priority order) of the Authority in developing or reviewing food regulatory measures and variations of food regulatory measures are (a) protection of public health and safety; (b) provision of information relating to food to enable consumers to make informed choices; and (c) prevention of misleading or deceptive conduct [36]. The Act also requires FSANZ to consider trade and industry issues such as competitiveness and efficiency, along with alignment to both national and international food standards. Additionally, risk assessments need to be based on the best scientific evidence. During the review or development of the Code, FSANZ is required to have regard to policy guidelines [36].

In 2003, the Ministerial Council developed its Policy Guideline on the Addition of Caffeine to Foods (Policy Guideline) with the aim of minimising the risk of dietary exposure to caffeine of at risk individuals in the population [2, 37]. In addition to the high order principles, the policy guidance statements include specific policy principles relating to caffeine risks [37, p. Ministerial Council Policy Guideline on the Addition of Caffeine to Foods] as follows.

*2003 Ministerial Council Policy Guideline on the Addition of Caffeine to Foods*

“*Other Principles*

1. Endeavour to limit the possible adverse effect of caffeine-containing foods on vulnerable subgroups of the population.
2. Ensure that the effect of caffeine additions to individual foods is considered in the context of the total diet.
3. Ensure the appropriate use of advisory statements on caffeine-containing foods in alignment with scientifically substantiated risk to vulnerable subgroups of the population.

Until further evidence becomes available, maintain the status quo (as currently in place in Australia) for caffeine regulation by

- Maintaining the current additive permissions for caffeine;
- Restricting the use of new products containing non-traditional caffeine rich ingredients (including guarana) to boost the caffeine content in other foods, beyond the current provisions for caffeine.

Caffeinated cola drinks and formulated caffeinated beverages will be permitted in accordance with the current standards. Foods, which naturally contain caffeine and have a long history of use and consumer awareness/association with caffeine, such as tea, coffee, and cocoa, are to be exempted from the labelling provisions and the use of these foods naturally containing caffeine to be added to other foods will continue to be allowed. Guarana, as a non-traditional food containing caffeine, will continue to have special labelling provisions outlined in the Food Standards Code.”

The review was ordered in response to ongoing public concern regarding the health implications of caffeinated beverage consumption, given the growth in the number and types of caffeinated products on the market [2]. The scope of the review included examination of current scientific evidence on the health effects of consuming caffeine (with a focus on the effects of dietary exposure of vulnerable population subgroups including children and adolescents); industry energy drink developments, for example, growth in the marketplace; and consideration of regulatory actions taken in other countries (e.g., for best practice and trade implications) [2]. Three policy options were proposed: to update; to maintain (keep the status quo); or to rescind the current Policy Guideline. After reviewing the evidence and following public consultation, a new Ministerial Policy Guideline-Regulatory Management of Caffeine in the Food Supply was endorsed on 27 June 2014 [38], with policy guidance statements, in addition to the high order principles [38, p. Ministerial Policy Guideline Regulatory Management of Caffeine in the Food Supply], as follows.

*2014 Ministerial Policy Guideline Regulatory Management of Caffeine in the Food Supply*

“*Specific Policy Principles.*  The regulatory management of caffeine in the food supply should

- be based on risk analysis ensuring consideration of general population and taking into account vulnerable population groups including children, adolescents, pregnant, and lactating women and caffeine sensitive consumers;
- consider exposure to caffeine from all dietary sources;
- be informed by emerging evidence and the regulation of caffeine in overseas jurisdictions.

*Additional Policy Advice*

 FSANZ is encouraged to work with research agencies to monitor caffeine consumption across the population, including consumption by vulnerable population groups.
 Regulatory management of caffeine in the food supply may include regulatory and non-regulatory risk management approaches.”

Despite the regulatory controls for FCB already in place, there is a high level of concern about sale of these beverages to children. In Australia, there have been calls to ban the sale of energy drinks to children and adolescents by the Country Women's Association with backing from the Australian Medical Association (AMA) [39]. Following the inaugural international energy drinks 2014 conference held in Australia, attendees released a statement highlighting evidence of adverse events associated with energy drink consumption and called for stronger regulatory approaches as the current measures had failed to protect the at risk subgroups, particularly children [40].

Measures proposed to reduce caffeine consumption in at risk subgroups include specifying a maximum retail unit volume, reducing the caffeine level in energy drinks, banning the sale of energy drinks to people under 18 years of age, restricting advertising and marketing of energy drinks to children and adolescents including events targeting this age group, and strengthening health warning advice to consumers [3, 13, 28, 30, 40, 41]. In response to concerns raised in Europe, Lithuania enacted a law banning the sale and advertising of energy drinks to people under eighteen years of age [42].

The findings of this current research suggest that the Australian and NZ government policy response is lagging behind public concern regarding the sale of caffeinated beverages to children. The Australian and NZ Food Regulation System identifies its stakeholders as including individual consumers, industry bodies, primary producers, food manufacturers, importers and retailers, public health organisations, consumer advocacy organisations, and community groups. There are opportunities during the development of standards for stakeholders to respond to public consultations. From time to time consumer research is undertaken by FSANZ and regular monitoring of public concern and opinion regarding contemporary food regulation issues is a role of government, such as the NMSS reported here.

Minimising the adverse events related to energy drink consumption is the policy goal and there are increasing calls for urgent action [40, 43, 44]. Strength of community opinion is considered when developing regulatory policy options and currently, across the WA population, adults support the use of food regulatory control of food labelling and advertising [45]. It is important that governments set limits to control the supply and promotion of food products with adverse health implications, particularly for vulnerable population groups. The Forum endorsement of the revised policy guideline on the regulatory management of caffeine in the food supply was, in part, in response to public concerns raised about risks associated with dietary exposure. This policy guideline will be used to guide the development or review of food standards relating to this important issue.

A limitation of this current research is that it is a cross-sectional study based on self-reported opinion which may be influenced by perceived social desirability and as the survey was conducted on an Australian population, care should be taken in generalising the findings. Further monitoring and surveillance of the contribution of specific foods to total caffeine intake is recommended, including coffee, tea, soft drinks, and energy drinks. Monitoring should report on mean caffeine intake, high acute and high chronic consumption levels, and the intake of population groups who are sensitive to caffeine, particularly children and adolescents.

## 5. Conclusion

The findings of high and growing level of community concern in WA regarding consumption of high caffeinated beverages to children, particularly by females, those living with children, and with increasing age, coupled with the worldwide public and scientific community concerns regarding adverse health effects for children and adolescents, are of interest to health promoters and regulators. In the current context of limited dietary intake data and the lack of an established acceptable daily intake level and considering the potential adverse health effects of acute or high intake, the precautionary principle applies. It is important that government maintain regulatory controls of formulated caffeinated beverages, including labelling, promotion, advertising, and sale of these beverages to children. The research findings suggest that the Australian and New Zealand government policy response is lagging behind public concern regarding the sale of caffeinated beverages to children.

## Figures and Tables

**Table 1:** Regulatory measures specific to the management of caffeine in food in Australia [22, 24, 25].

Regulatory measure	Key requirements
Standard 2.6.4 of the Code-Formulated Caffeinated Beverages	Must contain between 145 mg/L and 320 mg/L of caffeine and comply with labelling provisions disclosing nutrient composition including caffeine content (per serving and per 100 mL), along with daily usage and warnings that product is not suitable for children, pregnant, or lactating women

Standard 1.3.1 of the Code-Food Additives	Caffeine is approved as a food additive (flavour) in cola type drinks to a maximum amount of 145 mg/L

Standard 2.6.2 of the Code-Non-Alcoholic Beverages and Brewed Soft Drinks	Caffeine is prohibited as an ingredient in formulated beverage products

Standard 1.2.4 of the Code-Labelling of Ingredients	Caffeine must be included in the ingredient list where caffeine is added to the food

Standard 1.2.3 of the Code-Mandatory Warning and Advisory Statements and Declarations	Foods that contain guarana (rich in caffeine) are required to have an advisory statement on the label that the food contains caffeine. Other foods such as coffee, tea, and cocoa are not required to declare the presence of caffeine

New Zealand Food Safety Authority. * New Zealand Food (Supplemented Food) Standard 2013*	Foods that meet the definition of supplemented foods (excluding foods that meet the definition specified in Standard 2.6.4 of the Code) may contain caffeine for purposes other than as an additive. If containing more caffeine than is required to achieve a technological function under conditions of Good Manufacturing Practice the label on the package of supplemented food must include caffeine content (per serving and per 100 mL), along with daily usage and warnings that product is not suitable for children, pregnant, or lactating women Guarana may be added to a supplemented food, with the restriction that the label must include an advisory statement that the food contains caffeine

**Table 2:** Sample demographics of Nutrition Monitoring Survey Series, Western Australia, 2009 and 2012.

	2009 *n* = 1284	2012 *n* = 1548	Total *n* = 2832	Weighted^a^ %
Gender				
Female	830	1005	1,835	49.2
Male	454	543	997	50.8
Age groups				
18–24 years	71	66	137	15.6
25–34 years	180	144	324	22.5
35–44 years	340	377	717	22.7
45–54 years	356	466	822	21.6
55–64 years	337	495	832	17.7
Area of residence				
Metropolitan	965	1011	1976	79.3
Remote (Kimberley and Pilbara)	29	82	111	3.6
Rural	290	455	745	17.1

^a^Percentages were weighted for probability of selection and adjusted by age, sex, and geographic area to the 2011 Estimated Resident Population of Western Australia.

**Table 3:** Prevalence of how concerned participants are about the sale of high-caffeine drinks to children 12 years or younger, NMSS, 2009 and 2012.

	Total	2009	2012	*p* value^b^
	%	% [95% CI]	% [95% CI]	
Original categories (*n* = 2192)				.008
Not very concerned	5.3 [4.0, 7.1]	6.3 [4.4, 8.9]	3.5 [2.1, 5.7]	
Somewhat concerned	5.9 [4.5, 7.9]	6.1 [4.3, 8.4]	5.7 [3.3, 9.7]	
Neither unconcerned or concerned	2.9 [2.1, 4.0]	3.4 [2.3, 5.0]	2.0 [1.2, 3.4]	
Quite concerned	14.9 [12.9, 17.1]	17.1 [14.4, 20.2]	10.8 [8.2, 13.9]	
Very concerned	70.3 [67.7, 72.9]	66.5 [63.0, 69.8]	77.4 [73.0, 81.3]	
Do not know	0.6 [0.3, 1.1]	0.6 [0.3, 1.4]	0.5 [0.2, 1.4]	
Combined categories (*n* = 2122)^a^				.003
Not very concerned	5.5 [4.1, 7.3]	6.6 [4.6, 9.3]	3.6 [2.2, 5.8]	
Somewhat/quite concerned	21.7 [19.2, 24.3]	24.2 [21.0, 27.7]	17.1 [13.4, 21.5]	
Very concerned	72.8 [70.2, 75.4]	69.3 [65.7, 72.6]	79.4 [74.8, 83.3]	

^a^Excluded participants who said “neither unconcerned or concerned” and “don't know.”

^b^ *p *values were derived from a survey design-based Pearson chi square test.

**Table 4:** Factors related to how concerned the participants are about sale of high-caffeine food to children 12 years old or younger, NMSS, 2009 and 2012.

	How concerned the participants are about sales of high-caffeine food to children (from “not very” to “very” concerned) OR [95% CI]
Survey year	
2009	1.00
2012	1.38 [0.88, 2.17]
Gender	
Male	1.00
Female	2.11 [1.44, 3.10]^*∗∗∗*^
Age (years)	1.04 [1.02, 1.05]^*∗∗∗*^
Living with children	
No	1.00
Yes	2.16 [1.51, 3.09]^*∗∗∗*^
Residential area	
Metro	1.00
Remote (Kimberley and Pilbara)	0.55 [0.31, 0.99]^*∗*^
Rural	1.21 [0.82, 1.78]

^*∗*^ *p* < .05; ^*∗∗∗*^ *p* < .001. Results are odds ratio [95% confidence interval] from an ordinal logistic regression. The outcome variable is on a five-point Likert scale, from “not very concerned” (1) to “very concerned” (5).
